# Enhancing automated vehicle identification by integrating YOLO v8 and OCR techniques for high-precision license plate detection and recognition

**DOI:** 10.1038/s41598-024-65272-1

**Published:** 2024-06-22

**Authors:** Hanae Moussaoui, Nabil El Akkad, Mohamed Benslimane, Walid El-Shafai, Abdullah Baihan, Chaminda Hewage, Rajkumar Singh Rathore

**Affiliations:** 1 Engineering Systems and Applications Laboratory, National School of Applied Sciences, Sidi Mohamed Ben Abdellah University, Fez, Morocco; 2 Laboratory of Industrial Techniques (LTI), EST of Fez, Sidi Mohamed Ben Abdellah University, Fez, Morocco; 3 Security Engineering Lab, Computer Science Department, Prince Sultan University, 11586 Riyadh, Saudi Arabia; 4 Department of Electronics and Electrical Communications Engineering, Faculty of Electronic Engineering, Menoufia University, Menouf, 32952 Egypt; 5 Computer Science Department, Community College, King Saud University, 11437 Riyadh, Saudi Arabia; 6 Department of Computer Science, Cardiff School of Technologies, Cardiff Metropolitan University, Llandaff Campus, Western Avenue, Cardiff, CF5 2YB UK

**Keywords:** Deep learning, Yolo v8, Image segmentation, Character recognition, OCR, Thresholding, Morphological operation, Computer science, Information technology, Software

## Abstract

Vehicle identification systems are vital components that enable many aspects of contemporary life, such as safety, trade, transit, and law enforcement. They improve community and individual well-being by increasing vehicle management, security, and transparency. These tasks entail locating and extracting license plates from images or video frames using computer vision and machine learning techniques, followed by recognizing the letters or digits on the plates. This paper proposes a new license plate detection and recognition method based on the deep learning YOLO v8 method, image processing techniques, and the OCR technique for text recognition. For this, the first step was the dataset creation, when gathering 270 images from the internet. Afterward, CVAT (Computer Vision Annotation Tool) was used to annotate the dataset, which is an open-source software platform made to make computer vision tasks easier to annotate and label images and videos. Subsequently, the newly released Yolo version, the Yolo v8, has been employed to detect the number plate area in the input image. Subsequently, after extracting the plate the k-means clustering algorithm, the thresholding techniques, and the opening morphological operation were used to enhance the image and make the characters in the license plate clearer before using OCR. The next step in this process is using the OCR technique to extract the characters. Eventually, a text file containing only the character reflecting the vehicle's country is generated. To ameliorate the efficiency of the proposed approach, several metrics were employed, namely precision, recall, F1-Score, and CLA. In addition, a comparison of the proposed method with existing techniques in the literature has been given. The suggested method obtained convincing results in both detection as well as recognition by obtaining an accuracy of 99% in detection and 98% in character recognition.

## Introduction

**Figure 1:**
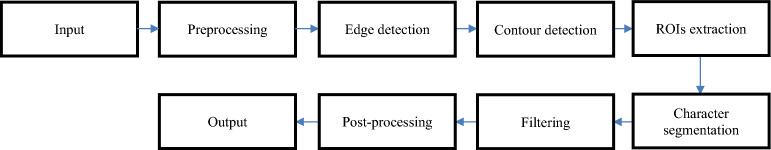
License plate detection flowchart.

Usually, license plate detection starts with object detection, in which a model is trained to locate license plates in an image or video frame. YOLO (You Only Look Once)^1^ and Faster R-CNN are popular object identification frameworks frequently utilized for this task. Afterward, images or frames are often preprocessed to boost contrast, emphasize features, and reduce noise to facilitate the detection model's ability to locate the plates. Moreover, detected zones are frequently filtered according to size, shape, or other factors to eliminate false positives. Afterward, edge and contour detection steps highlight edges and identify contours present in the image by employing several image processing techniques. Another important step in the license plate detection process is the regions of interest (ROIs) extraction, where the main purpose is to identify any potential region that might contain the license plate zone. The current process uses character segmentation and recognition techniques for separating and recognizing the present characters in the extracted license plate. Eventually, the number plate is refined using post-processing methods. Figure 1 below shows the license plate detection steps^2^.

**Figure 2:**
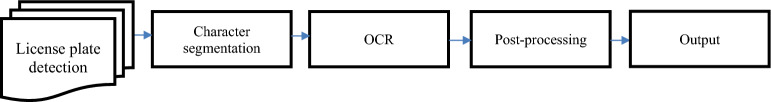
License plate recognition flowchart.

Subsequently, identifying the characters on the license plate comes next after it has been recognized, this is referred to as optical character recognition (OCR)^3^ for license plates. The characters on the plate are split from the identified plate region to separate individual characters or digits. nonetheless, the fragmented characters are recognized using an OCR engine. One popular open-source OCR engine for text recognition is Tesseract OCR. Deep learning techniques and specialized OCR models^4^ can be trained on license plate datasets to increase recognition accuracy. Lastly, post-processing may be applied to the identified characters. This could include fixing mistakes, removing incorrect plates, and ensuring the output satisfies predetermined standards (such as license plate format). Figure 2 below shows the license plate recognition system, which contains the main blocks that should be added to the previous license plate detection process to recognize the characters in the number plate.

Moreover, a large dataset of annotations on license plate images is needed to train the detection and identification models. To ensure the models are robust, these datasets could contain a variety of license plate styles, fonts, and backgrounds. Though the recognition model is trained to recognize characters, the detection model is trained to identify regions on license plates. Afterward, a test dataset is used to assess the system's accuracy and efficiency to ensure it satisfies the necessary specifications and can handle real-world situations. Eventually, a more extensive system, like parking management, security, or traffic management systems, incorporates the detection and recognition components. However, it's important to remember that different license plate techniques, lighting, and environmental circumstances might make detecting and recognizing license plates difficult.

For this reason, creating precise and dependable systems requires robust algorithms and properly annotated information. When implementing such systems, privacy and legal issues also need to be considered. To overcome these difficulties, ALPR systems usually employ deep learning models, image preprocessing, image enhancement, and data augmentation. Furthermore, ongoing research and development are necessary to increase the precision and resilience of license plate detection and identification systems. Accordingly, the proposed method aims at three essential axes: detection, recognition, and assigning each license plate to its original country. The vision of this method is promising and has long-term benefits in many areas, such as security. On the other hand, it's easy to implement and gives satisfactory results.

The rest of the article will be organized as follows, starting with a related work section, where the most relevant previous works in the literature have been gathered. Additionally, a detailed clarification of the proposed method is given in the third part of the article, where the methods used to create the proposed technique were explained point by point, as well as the evaluation metrics and the obtained results. Eventually, a conclusion and future work section is given.

## Related works

In computer vision and image processing, license plate detection and recognition (LPR)^5^ aims to extract license plates from images or video streams, recognize the characters on the plate, and then process the extracted information. Several methods and approaches have been developed via substantial research and development in this field to increase efficiency and accuracy. Therefore, in the literature, two main approaches exist for license plate detection (LPD) which are the traditional techniques, and the deep learning methods The first approach uses contour analysis, thresholding, and edge detection to find the license plate region in an image. As for the second technique, CNNs^6^ remains a popular choice for detecting license plates. Popular real-time license plate identification designs are SSD which refers to Single Shot MultiBox Detector^7^ and YOLO. On the other hand, license plate recognition uses optical character recognition that is used to identify the characters on a license plate once the region containing the plate has been identified. Commonly used tools include Tesseract OCR and commercial programs like ABBYY FineReader. Or, it employs deep learning-based optical character recognition such as CNNs and RNNs^8^, that have been used to ameliorate character recognition accuracy. Moreover, several methods have been proposed in the literature, here are some of them:

The authors of the paper (9)^9^ presented a method for independently predicting locations; by examining context information, the system produces smoother and more precise detection. It obtained a Hmean of 0.73, a recall of 0.71, and a precision of 0.74.

It is shown in paper (10)^10^ that by obtaining high-quality visual data, a pipeline based on convolutional neural networks can enhance text identification and recognition performance. This study employed a pre-trained ResNet-50 network to extract low-level visual features from ImageNet and SynthText.New and improved ReLU layer (new.i.ReLU) blocks are also part of the proposed structure. These blocks have good text component identification capabilities even on curved surfaces and a large receptive field. A new, improved inception layer can produce more effectively a broadly varying-sized text than a linear set of convolution layers.

In the article (11)^11^, the authors provide ReLaText, a novel approach for text detection. It works by redefining text identification as a visual link detection issue. Using a "link" relationship, they first tackle the difficult text-line grouping problem to illustrate the efficacy of this unique formulation.

An improved YOLOv3 network-based scene text detection algorithm is used in the research (12)^12^ on scene text detection. First of all, since YOLOv3 relies on the network of the Darknet53 backbone, which has several layers and is unable to train rapidly for a single detection target, this study proposes a technique by replacing the Darknet53 with the Darknet19. Second, the multi-scale detection of the original network was retained, and three different-sized anchors were employed to forecast the bounding boxes.

In the paper (13)^13^, the authors of this work present TextField, a novel text detector for recognizing usual scene texts. Particularly, when each text point should have an orientation field moving away from the closest text boundary. A fully convolutional neural network is used to learn this direction field, which is then represented by a 2D vector image. Unlike typical segmentation-based approaches, it stores the direction information required to discriminate between surrounding text instances as well as the binary text mask.

The authors of the research (14)^14^ offer an accurate text region representation method for text localization in scenes. Initially, text suggestions are extracted using a text region proposal network from an input image. A refinement network is then used to verify and enhance these suggestions.

In paper (15)^15^, the authors directly train a cross-modal similitude between each text instance from the natural images and the query text. In particular, they developed an end-to-end trainable network tuned for both scene text detection and cross-modal similarity learning.

The study in the paper (16)^16^ presents the Pixel Aggregation Network that the authors refer to as an accurate and efficient arbitrary-shaped text detector. It consists of a learnable post-processing component and a segmentation head with a low computing cost.

In the study (17)^17^, the authors developed a system to test the security of Hindi CAPTCHAS. For this, k-nearest neighbors, support vector machines, and random forest classifiers are used to crack ten distinct colored CAPTCHAs. Two-color schemes have a 90% breaking rate, whereas multi-color schemes have a 93% breaking rate.

In the paper (18)^18^, The authors proposed a technique to evaluate the security of CAPTCHAs based on Devanagari scripts. They selected five distinct monochrome and five grayscale CAPTCHAs for security testing. They produced six different kinds of features for these segmented characters and obtained segmentation rates ranging from 88.13 to 97.6% using these approaches. They employed three classifiers for comparative studies in their categorization process, using k-nearest Neighbor (k-NN), Support Vector Machine (SVM), and Random Forest. They attained a breaking rate of 73–93% for grayscale schemes and 66–85% for monochrome designs.

In the study (19)^19^, the authors suggest a brand-new module Multi-Domain Character Distance Perception to create a position embedding that is both semantically and visually connected. Using the cross-attention method, MDCDP queries both visual and semantic information using the location embedding. They created CDistNet, which guides an increasingly accurate distance modeling by stacking several MDCDPs. Table 1 summarizes the methods in the related works section.

**Table 1:** Findings of the literature.

The paper	Proposed method	Results
Paper (9)^9^	A pipeline based on convolutional neural networks is shown to extract high-level visual features and enhance the effectiveness of text detection and recognition	Recall: 0.71 Precision: 0.74
Paper (10)^10^	Text detection is formulated as a visual relationship identification problem by authors presenting a novel arbitrary-shaped text detection method called ReLaText	The authors conducted extensive tests on five scene text identification benchmark datasets to assess the effectiveness of their proposed ReLaText. To ensure that their results were comparable to those from other techniques, they adhered to the evaluation protocols outlined by the authors
Paper (11)^11^	An improved YOLO v3 for text identification algorithm is used in this study, focusing on scene text detection	The YOLOv3-Darknet19 and YOLOv3-Darknet53 algorithms are compared to assess their methodology. The results indicate that the Darknet19 network's loss is reduced quickly, the data swings less, and the final constant value minimizes
Paper (12)^12^	The authors provide TextField, a novel text detector that recognizes atypical scene texts	The experimental findings demonstrate that the suggested method performs significantly better on two curved text datasets than the cutting-edge techniques (28% and 8%)
Paper (13)^13^	This paper's authors provide a scene text identification technique that uses adaptive text region representation	Test results on five benchmarks: TotalText, ICDAR2013, ICDAR2015, CTW1500, and MSRA-TD500 demonstrate that this approach performs state-of-the-art scene text detection
Paper (14)^14^	The authors of this research directly learn a cross-modal resemblance along with a query text and every text occurrence using natural images. To be more precise, they created a fully trainable network by simultaneously improving the processes of cross-modal similarity learning and scene text detection	To assess the performance of their proposed method, they test their technique on three standard datasets to prove the effectiveness of their suggested approach
Paper (15)^15^	The authors of this research offer the Pixel Aggregation Network (PAN), a low-cost computational segmentation head and learnable post-processing for an accurate and efficient arbitrary-shaped text detector	F-measure on CTW1500 of 79.9% at 84.2 FPS
Paper (16)^16^	The authors provided a summary of the metrics and tools used for OCR evaluation and outlined two sophisticated applications for the output of OCR	For the experiment section, using two separate datasets and a variety of evaluation tools and criteria, the authors conduct an OCR evaluation experiment
Paper (17)^17^	The authors developed a system to test the security of Hindi CAPTCHAS	Two-color schemes have a 90% breaking rate, whereas multi-color schemes have a 93% breaking rate
Paper (18)^18^	The authors suggested a technique to evaluate the security of CAPTCHAs based on Devanagari scripts. For security testing, they selected five distinct monochrome and five distinct greyscale CAPTCHAs	The authors obtained segmentation rates ranging from 88.13% to 97.6%, a breaking rate of 73% to 93% for greyscale schemes and 66% to 85% for monochrome designs
Paper (19)^19^	the authors suggest a brand-new module named Multi-Domain Character Distance Perception (MDCDP) to create a position embedding that is both semantically and visually connected	To assess the performance of their proposed method, the authors compared CDistNet with nineteen other techniques published between 2017 and 2022

## Research gaps

Recent advancements in deep learning, machine learning, and computer vision techniques have contributed to enormous advances in license plate detection and recognition research. There are still some research gaps in this field, such as variable weather, occlusions (partially visible plates due to obstacles or barriers), and lighting conditions. Additionally, current algorithms frequently concentrate on standard license plate formats in particular areas, including alphanumeric plates. Moreover, while specific algorithms perform well when applied to novel datasets or real-world circumstances, their accuracy may decrease when used on other datasets or in highly regulated contexts. Furthermore, efficient algorithms with real-time processing capabilities are required, especially for platforms with limited resources, such as mobile devices and embedded systems. Therefore, by filling these research gaps, license plate detection and recognition systems will function better and be more reliable. They will also be widely used in various applications, such as parking and traffic management, law enforcement, and vehicle identification.

## The proposed method

**Figure 3:**
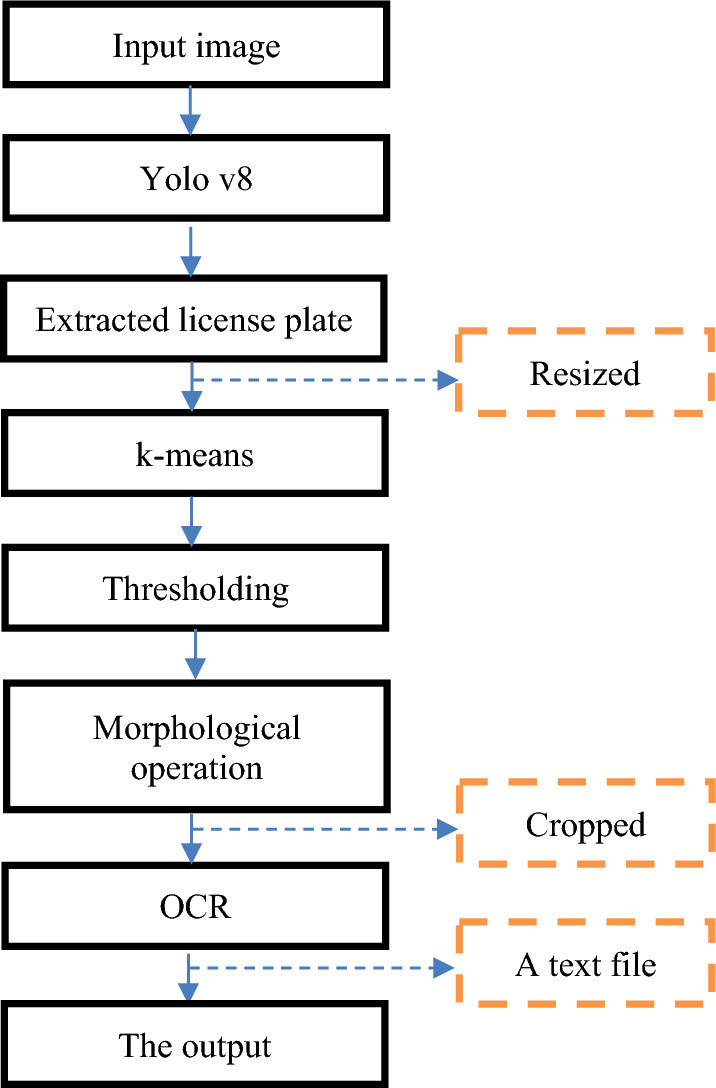
The proposed method flowchart.

This section discusses the proposed method's central parts, starting with describing the created dataset. Afterward, Yolo v8 detects the license plate in the input image. The detected plate will then be resized and enhanced by using some of the image processing techniques that play the preprocessing stage, such as k-means clustering, thresholding, and morphological operations. This step is crucial when achieving good accuracy in the character recognition part, especially with the noise that may occur during edge detection. Subsequently, the OCR algorithm will be applied to recognize the characters in the image. Afterward, a text file is generated containing only the essential part of the plate that will indicate the car's original country. The suggested method is shown in detail in Figure 3.

## Dataset

To train Yolo v8, a new dataset was created by gathering 270 images from the internet; these images are publicly available and can be downloaded without restriction. Furthermore, this dataset contains cars taken from different angles and under various lighting conditions.

Moreover, to guarantee that a dataset is representative, diversified, and appropriate for the intended purpose, selecting images for it requires careful consideration of several factors, including diversity, by collecting images with different light conditions, scenes, and viewpoints. On the other hand, the created dataset includes images with various instances of the target object, the license plate. Another important criterion is the ethical considerations when selecting and downloading only images that respect people's privacy.

However, these particular requirements may change based on the project's objectives. Eventually, the data was annotated accurately.

**Figure 4:**
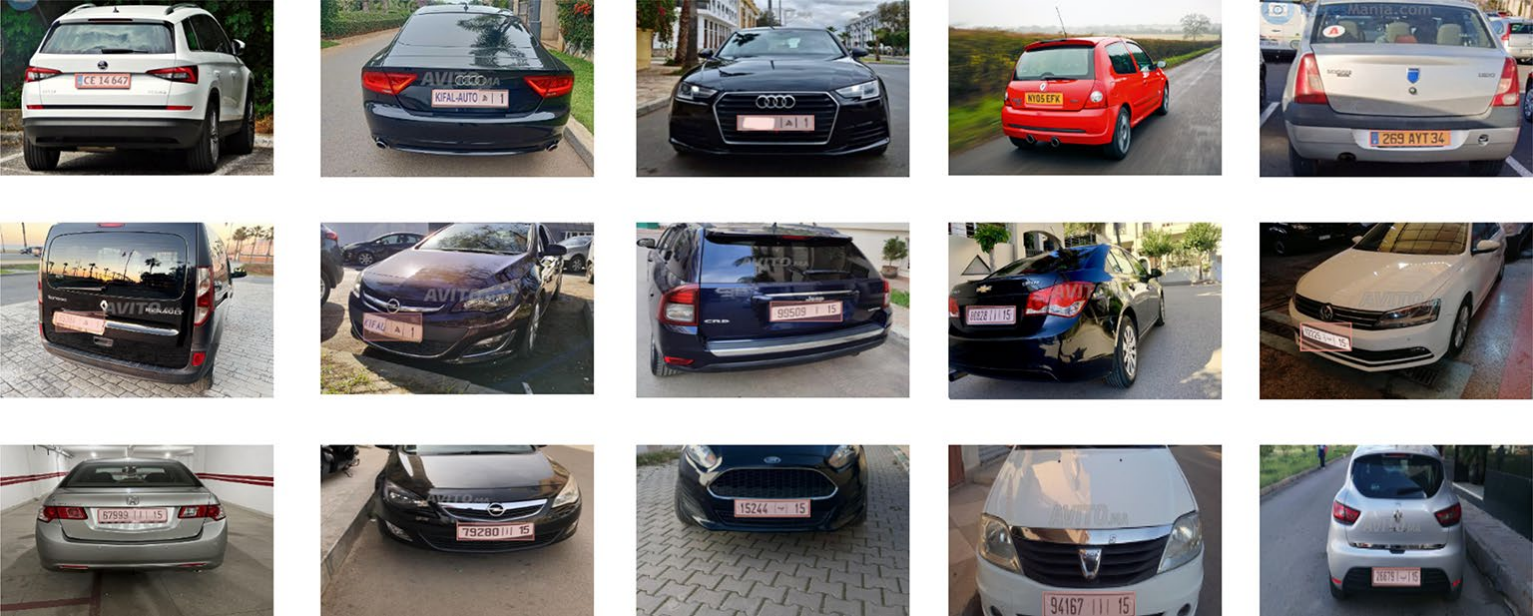
The annotated dataset.

Afterward, the CVAT tool was used to annotate the data and generate the annotations. Moreover, the dataset was divided into three categories: train, validation, and test. Figure 4 demonstrates the annotated dataset. Furthermore, at the end of the manuscript, a declaration statement containing the public sources from which the images were downloaded is given.

As mentioned earlier, the CVAT annotation tool was chosen to annotate the data used for this study. Here’s a slight clarification about the CVAT annotation process and the steps to follow:

- *Step 1* Create a free account on the CVAT platform; https://www.cvat.ai/
- *Step 2* Create a new task
- *Step 3* Enter the necessary label(s)
- *Step 4* Upload and submit the data
- *Step 5* Click on the “Job” section and start annotate
- *Step 6* When done labeling, export the labeled dataset to a specified format

## The inter-annotator agreement

In image processing, inter-annotator agreement describes the level of consistency or agreement across many human annotators that independently label or annotate the same collection of images. This is especially crucial for tasks involving subjective interpretation, like segmentation, object detection, and image classification. Moreover, several annotators may work together to classify images in image processing to provide a ground truth dataset that can be used to train and assess machine learning models. The inter-annotator agreement helps evaluate the consistency and dependability of annotations made by several human annotators. Inter-annotator agreement is typically measured using several metrics, such as Cohen's Kappa coefficient, Fleiss' Kappa, Jaccard index, and dice coefficient. In this study, the annotation process was conducted by two authors (two annotators) using the same annotation tool mentioned earlier.

Moreover, since only one class label is needed in this study, referred to as the license plate label, it was unsuitable to use Cohen's Kappa Coefficient, which requires two or more class labels. Additionally, it needed to be better to implement Fleiss' Kappa, which requires more than two annotators. Instead, the Jaccard index, as well as the Dice coefficient, were used to measure the inter-annotator agreement between the two labeled sets of images given by the two annotators. Therefore, in this case, the Jaccard index determines the IoU by dividing the union of these regions by the intersection of the regions where both annotators label the license plate. To indicate agreement, the overlap between the places where annotators located license plates will be measured by the IoU metric. On the other hand, the Dice Coefficient can be computed similarly to the Jaccard Index by dividing the total size of each zone by twice the intersection of the areas where both annotators label the license plate. Between the license plate regions that the annotators have determined, the Dice Coefficient measures the agreement in terms of overlap.

The obtained values in Table 2 show that the annotators have given a similar annotation which has afterward influenced positively the accuracy of the model that has been used for the license plate detection.

**Table 2:** Results of inter-annotator agreement metrics.

The inter-annotator agreement metrics	Jaccard index	Dice coefficient
The obtained values	99.95%	99.99%

## Yolo v8

Yolo v8 is Ultralytics' most recent iteration of YOLO. With new features and enhancements for improved performance, flexibility, and efficiency, YOLOv8^20^ is a cutting-edge model that builds on the success of earlier iterations. YOLOv8 supports all everyday visual AI tasks, such as tracking, segmentation, posture estimation, detection, and classification. Because of its adaptability, users can use YOLOv8's features in multiple applications and domains. "You Only Look Once" or "YOLO"^21^ is a well-known and significant object recognition framework in computer vision and deep learning. Yolo was developed to increase object identification in real-time application speed and accuracy. It deviates from conventional object detection techniques by framing object identification as a regression problem and making predictions for object bounding boxes and class labels in a single forward run of a neural network. The following are some of the main ideas and characteristics of the YOLO algorithm^22^, ^23^:

Single-pass Detection: YOLO predicts bounding boxes and class probabilities for objects by processing the entire image or video frame in a single pass. Several additional object-detecting systems, on the other hand, employ multi-stage procedures.

Grid-based Approach: YOLO creates a grid out of the input image, and each grid cell has to guess what kind of object is inside it. YOLO forecasts bounding boxes and associated class probabilities for every cell.

Bounding Box Predictions: Yolo predicts the bounding box coordinates surrounding observed items. It forecasts the bounding boxes' height (h), width (w), and center coordinates (x, y). The forecasts are based on the grid cell's dimensions.

Class Predictions: YOLO further makes predictions about the likelihood that each object it detects will fall into a particular class, such as "car," "person," or "dog." This enables YOLO to categorize items and detect them.

Non-Maximum Suppression: YOLO uses non-maximum suppression (NMS) after formulating predictions to eliminate low-confidence or duplicate detections. The final set of detected objects is refined with the aid of NMS.

**Figure 5:**
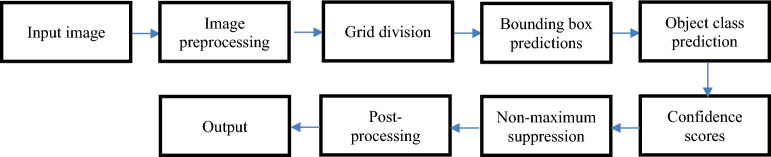
Yolo algorithm process.

**Figure 6:**
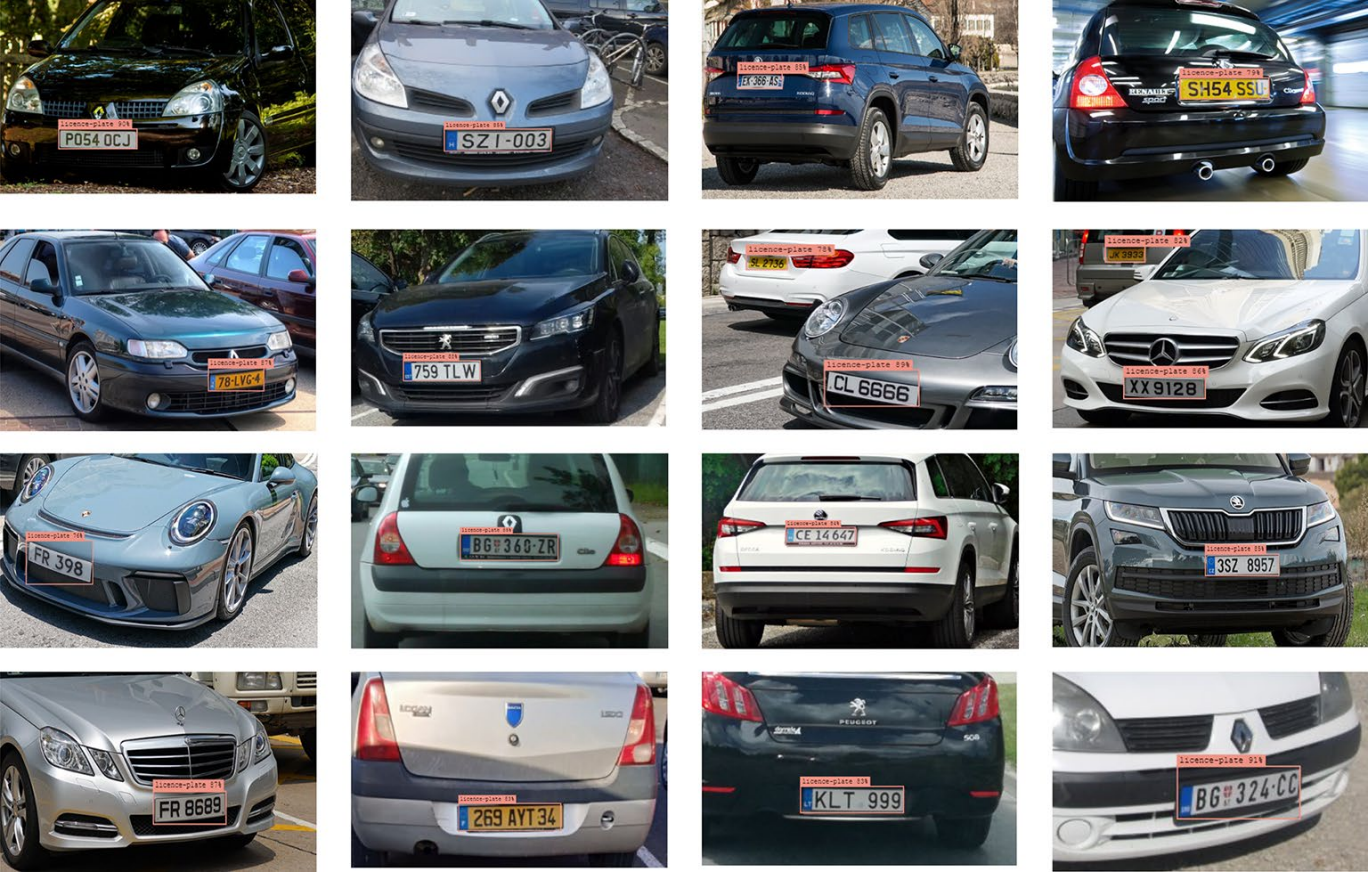
The detected license plates using Yolo v8.

The YOLO algorithm applies a set of steps to identify and find objects in images or video frames. The following Figure 5 shows the main steps in the YOLO algorithm:

Moreover, Yolo's widespread appeal in the computer vision world can be attributed to its exceptional accuracy in real-time object recognition. Researchers and engineers are still working on more improvements and iterations of the YOLO algorithm. Figure 6 below shows the obtained results after applying Yolo v8 to the created dataset:

Bounding box predictions are crucial to the success of YOLO in a variety of applications, from autonomous navigation to surveillance, as they enable precise localization, size estimate, as well as immediate evaluation for several objects in images. Moreover, these arguments demonstrate why YOLO v8-based license plate identification systems require bounding box predictions. All of these processes’ verification, tracking, and alphanumeric recognition are made easier by YOLO's ability to locate and identify license plates in a variety of settings and configurations. These processes are essential for automated systems used in traffic control, law enforcement, and vehicle-related services. YOLOv8 offers enhancements to the developer experience as well as architecture. In contrast to its predecessor, YOLOv8 includes:

1. A brand-new system for anchor-free detection.
2. Modifications to the model's convolutional blocks.
3. The mosaic augmentation that was used throughout the training was disabled before the last ten epochs.

Moreover, YOLOv8 includes modifications to enhance the model's development experience. To begin with, the model is now provided in the form of a library that was usually added to the Python code.

The reason behind choosing the Yolo model over the other deep learning techniques is that Yolo is widely known for its ability to process information in real-time. Moreover, it is appropriate for applications where low latency is essential, like license plate recognition in traffic surveillance systems, because it can process images as well as videos quickly. Furthermore, YOLO uses a one-pass architecture, the entire image is processed by the neural network during a single forward pass. This contrasts with several object detection techniques that make use of numerous passes and region proposal networks (RPNs).

On the other hand, and because of its excellent generalization capabilities across several object categories, YOLO is a good choice for a variety of detection tasks, including the identification of license plates. Its architecture can efficiently handle a wide range of object sizes and aspect ratios, also, it successfully strikes a balance between precision and speed. Eventually, for every bounding box prediction, YOLO assigns a score that indicates the probability that the bounding box has an object of interest. This improves the model's detection of meaningful objects and assists in filtering out false positives.

## Preprocessing

To increase the recognition system's accuracy and resilience, preprocessing the identified license plate area for character recognition is essential. For this reason, and by using k-means clustering and thresholding, the image is first divided into separate areas according to the intensity of its pixels, in this stage, pixels with comparable brightness are clustered together to help distinguish the characters from the background. Thereafter, a threshold is applied to binarize the image, where character pixels with intensity values exceeding the threshold are categorized as foreground, and background pixels are identified as pixels having intensity values below the threshold. Eventually, a morphological technique, which is the opening operation, is applied to enhance the binary image's quality and eliminate minor noise. Moreover, to eliminate unnecessary background and to concentrate just on the area with the characters, the identified license plate region was cropped.

## K-means algorithm

**Figure 7:**
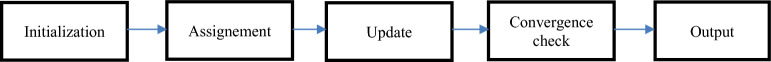
K-means clustering process.

*K*-means clustering divides data points into *K* unique, non-overlapping subgroups or clusters^24–26^. Their centroids, or centers, are what characterize these clusters. To find patterns and put related data points together, the technique is frequently used in data analysis, data mining, image segmentation^27–29^, and associated domains. The following Figure 7 presents the required steps that the *K*-means clustering algorithm uses:

- *Step 1* Select *K* initial centroids first. These centroids can be chosen at random from the data points or positioned with more consideration for context.
- *Step 2* Determine the closest centroid for every data point by applying a distance measure, most commonly the Euclidean distance. In this stage, K clusters are created, and every data point is assigned to the cluster that has the closest centroid.
- *Step 3* Determine the new centroids for every cluster by averaging all of the data points that belong to that cluster. These new centroids represent the core of each cluster.
- *Step 4* Assess whether there have been any notable changes to the centroids from the prior iteration. Subsequently, moving to the next stage if the centroids have stabilized and the algorithm has converged.
- *Step 5* The *k* cluster centroids and the assignment of every data point to a cluster are the ultimate results of the *K*-means algorithm. This information can be used to evaluate the data and comprehend the clusters' structure.

It's crucial to remember that the original centroids chosen can have an impact on how well the clustering outcome turns out. It is usual practice to run *K*-means numerous times with different initializations and choose the optimal outcome according to a criterion like minimizing the total within-cluster variance^30^. This is because random initialization can occasionally result in inadequate solutions.

The *k*-means clustering mathematical formula is presented below:

1 $$J=\sum_{j=1}^{k} \sum_{i=1}^{n} ||{x}_{i}^{(j)}-{c}_{j}|{|}^{2}$$

where

J: The objective function

N: The number of the case

K: The number of the cluster

${c}_{j}$: The cluster’s j centroid.

$${x}_{i}^{(j)}$$: The I case.

*k*-means clustering has been used as a part of the whole process of the proposed method for three main reasons:

- To distinguish the foreground (the license plate characters) from the background in the image.
- Regions related to characters can be identified and segmented based on color using k-means clustering. This helps get the image ready for the next stages of character recognition.
- Pixels in grayscale images can be grouped according to intensity using *k*-means clustering. This can help emphasize areas of the image that will probably contain characters or increase the contrast in the image itself.

For this research, several values for the number of clusters *k* were tested, with *k*=2 being selected upon achieving better results.

## Thresholding

Thresholding is a widely used image processing technique^31^ in binary images that is employed to separate important objects or features. The threshold value, which is a specific range of values or intensity level, must be determined to convert a grayscale image into a binary format, in which the value of a pixel above the threshold is assigned to one (white). and pixel values that are below the threshold will be set to zero (black). Here's a quick rundown of how thresholding works:

- *Step 1* The input image is usually converted to grayscale first. This streamlines the thresholding procedure by emphasizing intensity values over color.
- *Step 2* Selecting a Threshold. Selecting a Limit. It’s required to decide on an appropriate threshold value. The ideal threshold depends on the particular application and the characteristics of the input image. Popular strategies for threshold selection include Otsu's method, which uses image histograms to set thresholds automatically; manual selection; and adaptive thresholding, in which the threshold travels throughout the image to account for local variations in lighting and contrast.
- *Step 3* Operating Threshold. Once chosen, each pixel in the grayscale image is compared to the threshold value. If the intensity of the pixel is greater than or equal to the threshold, it is set to white; if not, it is turned to black. This results in a binary image where objects of interest are represented in white on a black backdrop.

**Figure 8:**
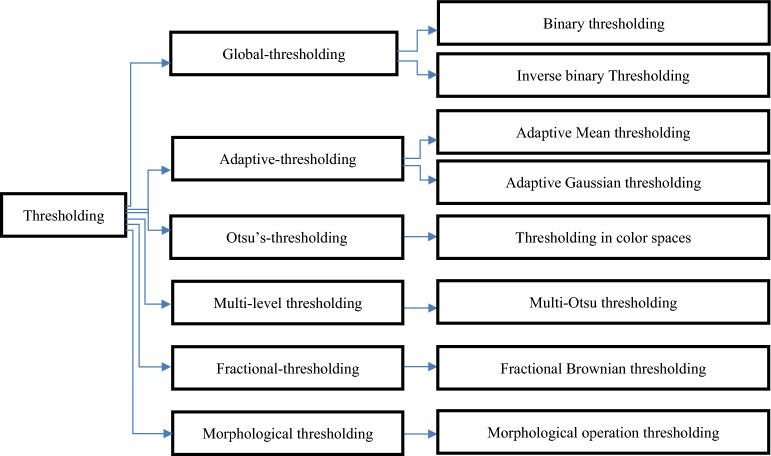
Thresholding algorithm types.

Figure 8 below presents some of the popular thresholding techniques^32^

The effectiveness of thresholding depends on several factors, including the input image's quality, the threshold value chosen, and the distinctive characteristics of the background and objects in the image. It can be necessary to carefully consider and test several thresholding strategies and threshold values before deciding on the optimal one for a given task.

The intensity levels of the pixels in an image are compared to a predetermined threshold value in the mathematical method for thresholding.

Let’s assume that t is the threshold value, and intensity (x, y) is the intensity value of a specific pixel at a given position (x, y) in the input image. The mathematical formula of thresholding^33^ can be written as below:

- Binary thresholding
  2 $$\left\{\begin{array}{cc}1& if intensity (x, y)>t\\ 0& otherwise\end{array}\right.$$
- Inverse binary thresholding
  3 $$\left\{\begin{array}{cc}0& if intensity (x, y)>t\\ 1& otherwise\end{array}\right.$$
- Adaptive mean thresholding
  4 $$\left\{\begin{array}{cc}1& if intensity (x, y)>t (x, y)\\ 0& otherwise\end{array}\right.$$
  Where t (x, y) is the mean of the values of the pixels around (x,y)
- Adaptive Gaussian thresholding
  5 $$\left\{\begin{array}{cc}1& if intensity (x, y)>t (x, y)\\ 0& otherwise\end{array}\right.$$
- To zero
  6 $$\left\{\begin{array}{cc}intensity (x, y)& if intensity \left(x, y\right) \ge t (x, y)\\ 0& if intensity \left(x, y\right) <t (x, y)\end{array}\right.$$

where t (x, y) is the weighted sum of the values of the pixels surrounding (x, y)

For this case study, and after trying several thresholding techniques, the most satisfactory results were achieved when applying the “To zero” thresholding type, more precisely when setting the threshold value to 180. Moreover, in the case of license plate detection and recognition, thresholding remains a crucial method since it streamlines the image processing process, making it easier to extract and identify the license plate's characters. Therefore, Through the enhancement of contrast, reduction of noise, and facilitation of the extraction of important information from the image, thresholding streamlines the image processing involved in license plate identification systems. Therefore, it acts as a prelude to bettering the precision and effectiveness of later processing phases, such as (OCR) and character segmentation.

## Morphological operations

Morphological operations^34^ are image processing techniques used to manipulate the shape and structure of objects in the image. These methods are very helpful for tasks like image segmentation, noise reduction, and feature extraction. Morphological methods usually use binary images or grayscale. Furthermore, the two most popular morphological processes dilation and erosion can be utilized singly or in combination to achieve a range of image-processing goals.^35^

- Dilation: In binary images, this is a morphological operation that enlarges the white regions or objects. All of the pixels that a tiny matrix or kernel, which serves as a structural element, scans across the image are colored white. The center of each structuring element is placed over a white pixel in the image at each position where it overlaps with it. Dilation can also be used to join dissimilar objects, thicken things, and fill in gaps. It can be utilized to enhance the notable attributes.
- Erosion is the opposite of dilation. It results in the white portions in a binary image shrinking or eroding. Erosion uses a structural element identical to dilation, but instead of changing the selected pixel to white if there is overlap, it only does so if all the pixels below it are white. If there is a black pixel below the structuring element, the target pixel is changed to black. Erosion can be used to remove little artifacts and disconnect related components, and thin objects.
- Opening: An opening operation is carried out by combining erosion and dilatation. It assists in removing little details and noises from an image whilst preserving the overall proportions and form of larger objects.
- Closing: A closing operation is the reverse of an opening activity. Erosion is the last step after dilatation. It is effective in filling up small gaps in items and connecting nearby components.
- Morphological gradient: This technique separates dilation from erosion in the given image. It highlights the edges of the objects in the image.
- Top Hat and Black Hat: These techniques involve subtracting the result of a closing or opening operation from the original image. The top-hat procedure draws attention to the lighter portions while the black-hat technique highlights the darker areas.
- Hit-or-miss transform: It’s a process that is used to recognize specific patterns or forms in binary images. This technique requires identifying similarities among the two predefined structuring elements in the image: one for the pattern in question and one for its counterpart.

Therefore, the opening technique was chosen for this case study to remove any potential noise from the finished product while maintaining the overall form of the characters. Moreover, artificial intelligence and image analysis frequently use morphological techniques for text extraction processes, image segmentation, object recognition, and other purposes. They can increase image quality, reduce noise in photos, and enhance features for further analysis. The size and form of the structuring element are determined by the specific image processing project at hand as well as the features of the image.

## OCR

Several document types, including PDF files, digital documents, and electronic photographs, can be transformed into editable and searchable data by using the OCR technology^36^. It is possible to extract, edit, and search for text in these computer files using OCR software and algorithms that evaluate the text and convert it into understandable machine code. The main parts and techniques of OCR are as follows^37^:

- *Step 1* Since an image is typically used as the given document for OCR, this step happens first. This entails making modifications to the picture, removing noise, and binarizing it (turning it into black and white). These procedures enhance character recognition input quality.
- *Step 2* OCR often needs to identify the portions of an image containing text. Text localization is the process of identifying the places where text is present.
- *Step 3* With multiple text lines or paragraphs, OCR systems need to divide the text into individual characters or words. Text segmentation refers to breaking down a text into its constituent parts.
- *Step 4* OCR systems often require to identification of the text-containing regions of an image. The process of text localization involves identifying the places where text is present.
- *Step 5* Fundamentally, each segmented character or word is recognized using optical character recognition (OCR), which transforms it into machine-readable text. Character recognition can be accomplished by a variety of techniques, including pattern matching, neural networks, and feature extraction methods.
- *Step 6* OCR software may utilize methods for post-processing after character recognition to improve error correction and text detection precision. This may include dictionary-based corrections, spell-checking, and context analysis.
- *Step 7* After identification, the text is made accessible in a way that machines can read such as plain text, searchable PDFs, or other kinds of documents. This allows users to edit, search, and save the content digitally.

Improvements in deep learning and machine learning have led to considerable achievements in OCR technology over time. High-accuracy OCR can be achieved by modern systems, even when dealing with intricate fonts, languages, and document layouts. There is open-source and commercial OCR software available, and OCR APIs are frequently integrated into different services and applications for automated data extraction and document processing.

To convert the characters from the image, different OCR techniques are provided, such as Tesseract OCR, Paddle OCR, Easy OCR, and Keras OCR. Moreover, the EasyOcr technique was chosen for three main reasons.

- Quick and effective: EasyOCR can process a lot of images in real time because it is designed to be quick.
- Simple to use: Python programs can readily incorporate EasyOCR into their code thanks to its straightforward interface.
- Good accuracy: Across a range of OCR criteria, EasyOCR has attained high accuracy.

## Yolo v8 and OCR integration

Depending on the use case and requirements, several approaches may be used to integrate Yolov8 along with OCR techniques in particular settings. YOLOv8 can be applied to real-time object detection. For this, the model must be trained using pertinent data to this field to incorporate this capability into a given space. For instance, the model needs to be trained on images of equipment and machinery unique to the manufacturing sector when wanting to recognize items in a manufacturing setting. Subsequently, text can be extracted from objects in an image using optical character recognition (OCR). Applications including document scanning, traffic sign translation, and license plate recognition can all benefit from this technology. Consequently, the results of applying OCR and YOLOv8 to an image can be combined in many ways based on the requirements. For instance, when recognizing license plates, the characters found by OCR can be linked to the cars found by YOLOv8 to determine who the owners of the cars are. On the other hand, in images and video streams, YOLO can recognize traffic indicators like stop signs, speed restriction signs, and traffic signals. Subsequently, OCR methods might be utilized to identify text or symbols on the signs, offering extra context for self-driving cars. Moreover, YOLO v8 and OCR techniques can be found in surveillance footage, where YOLO v8 can identify objects of interest like people, cars, or suspicious objects. Eventually, building intelligent systems that can detect and identify objects and text in images is made achievable by combining these techniques. This opens up a variety of applications in various industries, including document processing, retail, transportation, and surveillance. Typically, it involves preprocessing images, using YOLO v8 for object recognition, OCR technique to extract text, integrating the results, and optimizing performance.

## Experiments and results

**Figure 9:**
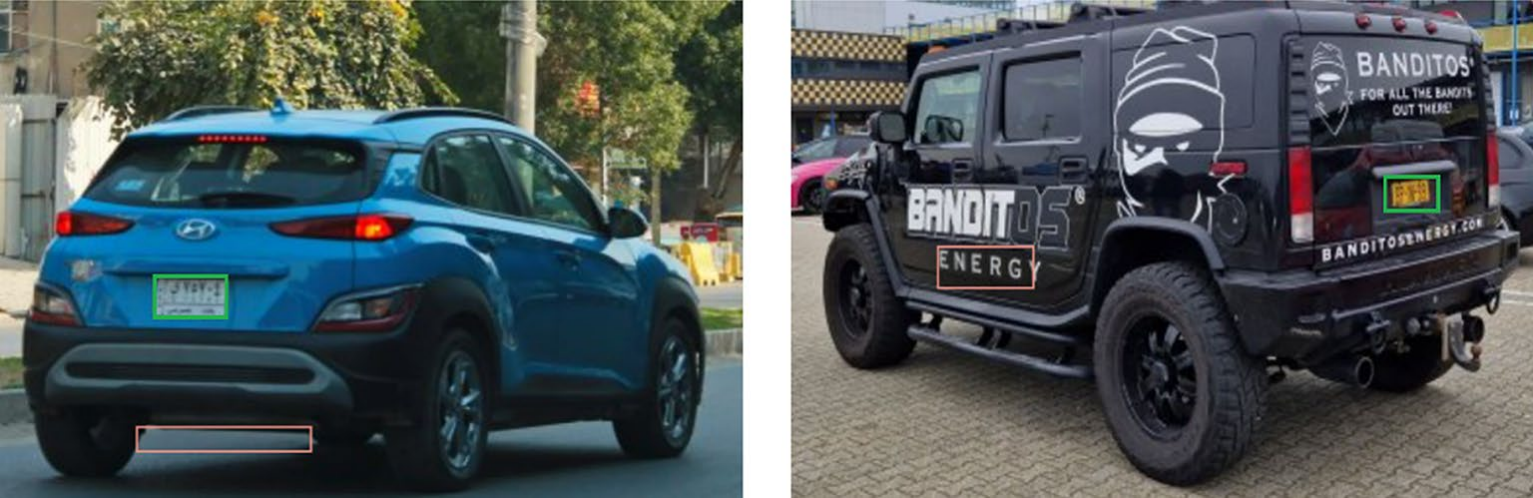
Examples of false positives. Red and green, respectively, outline the expected position as well as the ground truth.

Several evaluation metrics were employed, which are listed below, to evaluate the results of the suggested approach^38^. Therefore, when it comes to license plate detection, the terms "false positives" and "false negatives" have particular meanings associated with the detection system's accuracy. A false positive happens when the system misidentifies an area of an image as having a license plate when it doesn't. Stated differently, false positives are the results of the system mistakenly identifying a region that is not a license plate for a license plate. Furthermore, when a system is unable to identify the actual license plate area in an image, it results in a false negative for license plate detection. As a result, false negatives are the result of the algorithm missing or failing to detect a region that has a license plate. Additionally, testing the license plate detection on the created dataset yielded a higher recall and precision (99%), with only 2 false positives and no false negatives recorded. Figure 9 illustrates the 2 false positives obtained.

### Precision

Precision is often used to describe the degree of certainty in machine learning, statistics, and various domain labeling systems or prediction models. It measures the degree to which the system's positive predictions and actual positive events agree. The following formula is used to determine precision when dealing with binary classification, or a simple "yes/no" prediction:

7 $$Precision = \frac{True \,Positives}{False \,Positives+ True \,Positives}$$

The incidents that were correctly predicted to be positive are the real positives. Furthermore, cases mistakenly classified as positive are known as false positives. The obtained findings are presented in Table 3 below:

**Table 3:** Precision per epoch.

Precision	0.7429	0.8387	0.8909	0.9637	0.9641	0.9606	0.9768	0.9984	0.9990
Epochs	40	45	58	68	167	212	221	223	299

**Figure 10:**
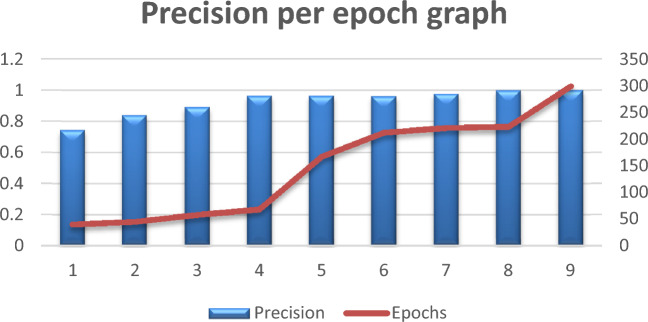
Precision per epoch graph.

Figure 10 below shows the graphic representation of Table 3:

From Table 3 and Figure 10 above, it’s noticeable that the precision has a consistent upward trajectory over all epochs and increases continuously. As training continues on the created dataset, it shows that the model is improving in accurately detecting positive cases. This indicates that the model is learning to produce more accurate positive predictions, which is generally a good indication.

### Recall

Recall, also known as sensitivity or true positive rate, represents a metric used in machine learning and statistics to evaluate a classification model's performance, particularly in binary classification settings. The model's recall measures its ability to recognize each relevant instance in the dataset. The mathematical formula of the recall metric can be written as follows:

8 $$Recall = \frac{True \,Positives}{False \,Negatives+ True \,Positives}$$

The obtained results are given in Table 4 below:

**Table 4:** Recall per epoch.

Recall	0.7709	0.4814	0.9444	0.9818	0.9815	0.9951	0.9976	0.9815	0.9948
Epochs	40	45	58	68	167	212	221	223	299

**Figure 11:**
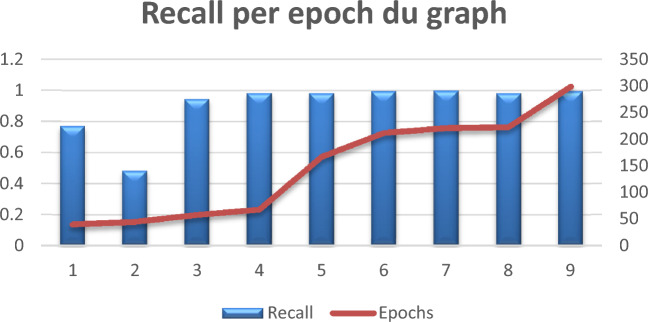
Recall per epoch graph.

Figure 11 below shows the graphic representation of Table 4:

In Table 4 and Fig. 11, a high recall per epoch is noticeable which indicates that the model's ability to distinguish true positive examples from all of the real positive instances in the dataset is either becoming better or changing over training, which means that the model is getting more sensitive to positive occurrences in the data, which is generally a good indication.

### F1-score

F1-score is a commonly used metric in computer learning and statistics that combines recall and precision into an individual number to provide a fair assessment of a model's performance, especially in binary classification problems. The F1-score mathematical formula can be written as follows:

9 $$F1-score = \frac{2\times Precision\times Recall}{Recall + Precision}$$

The obtained results are shown in Table 5 below:

**Table 5:** F1-score per epoch.

F1-score	0.7566	0.6116	0.9168	0.9726	0.9727	0.9775	0.9870	0.9898	0.9968
Epochs	40	45	58	68	167	212	221	223	299

**Figure 12:**
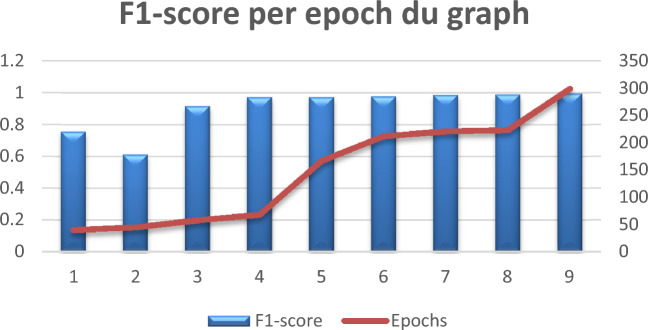
F1-score per epoch graph.

Figure 12 below shows the graphic representation of Table 5:

In Table 5 and Fig. 12, a high F1-score per epoch is noticeable, which means that the model is hitting a good balance between recall and precision. The F1-score is a statistic that takes into account both false positives and false negatives, as it is a harmonic average of precision and recall. it means that as training goes on, the model gets better at correctly classifying both positive and negative instances. This shows that the model has improved the trade-off between recall and precision.

### Accuracy

One commonly used metric to evaluate the overall accuracy^39^ of a predictive model is the Character-Level Accuracy. It calculates the proportion of each occurrence in the dataset that could have been predicted with accuracy. The mathematical formula of accuracy can be written as follows:

10 $$CLA=\frac{Number \,of \,correctly \,recognized \,characters}{Total \,number \,of \,characters}\times 100\%$$

In Table 6 below, the obtained accuracy for character segmentation is displayed:

**Table 6:**
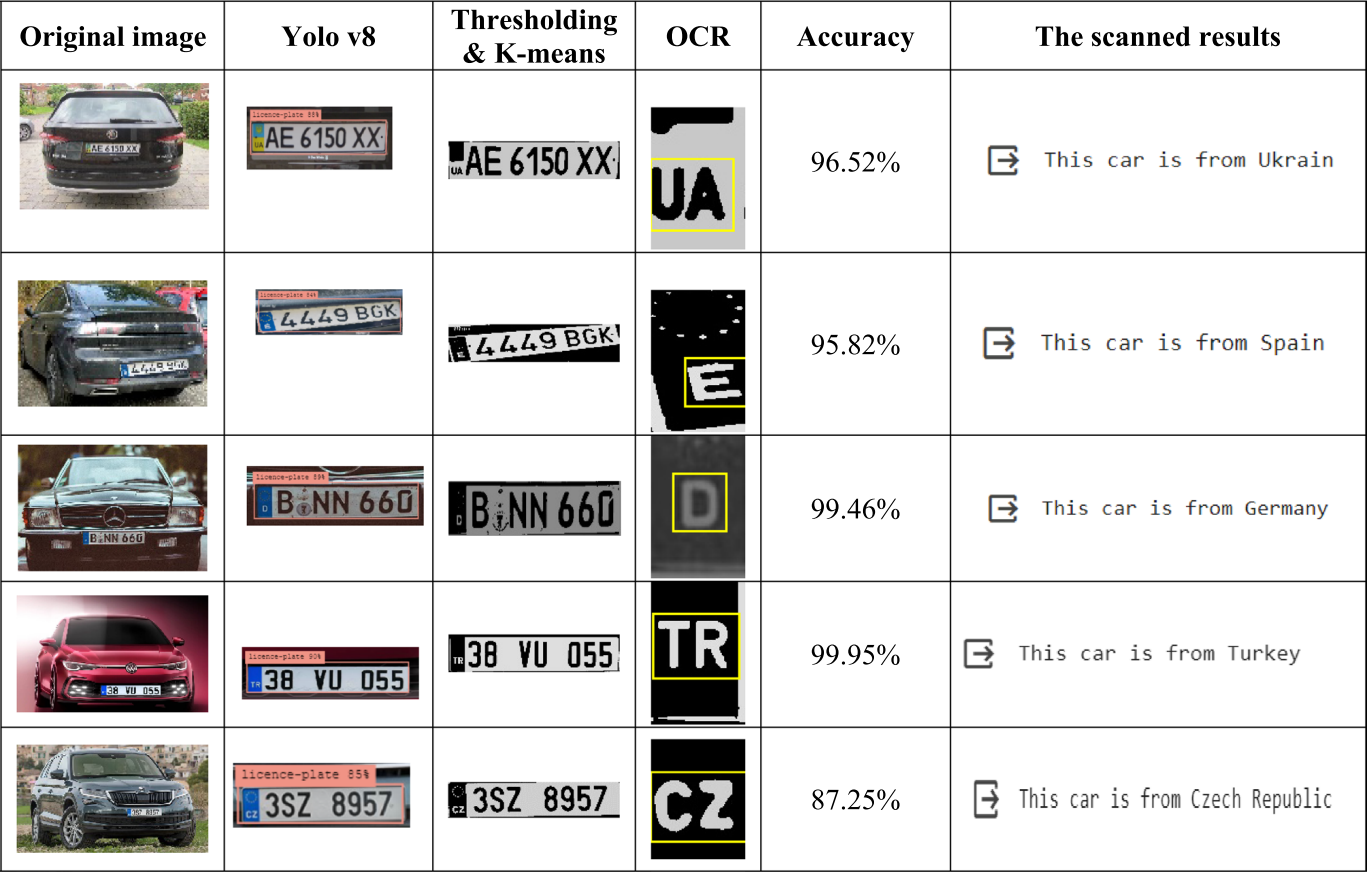
The obtained results.

From the obtained results, it’s seen that the proposed method gives satisfactory results in both detection and recognition of the text in the license plates when achieving an accuracy of 98% in the recognition and 99% in detection. The hybridization of Yolo v8 and the used image processing methods wasn’t arbitrary, these techniques were picked up carefully to have better results even in difficult scenarios where the lightning is bad or the angle at which the image was taken varies. Moreover, to have these results, many other techniques were tested before finding the suitable ones. Furthermore, a comparison with other methods that are given in Table 7 has proved the efficiency of the proposed method.

**Table 7:** Comparison of the proposed method with other techniques.

Methods	The proposed method (%)	Paper 1^40^ (%)	Paper 2^41^ (%)	Paper 3^42^ (%)
Detection process (F1-score)	99	97.9	96.09	65.5
Character recognition process (Accuracy)	99.95	97.5	–	87

In paper (1)^40^, the authors used the mask region convolutional neural networks (mask R-CNN) to detect the license plate. afterward, to segment the characters from the detected license plate, they used the Mask R-CNN-based method to classify characters and non-characters. In the paper (2)^41^ the authors used a hierarchical Convolutional Neural Network (CNN). The main idea is to use two passes of the same CNN to identify the license plate area. Afterward, a second CNN will be used to recognize the characters. In the paper (3)^42^, the authors proposed a system for detecting and recognizing license plates. For this, they pre-processed the input image using the Median filter in addition to histogram equalization. Afterward, they used Sobel edge detection to detect the license plate as well as labeling the obtained images and separate each object. Subsequently, to segment the characters they used the thresholding technique in addition to extracting the connected components. Finally, they employed the BPNN architecture to recognize the segmented characters.

**Figure 13:**
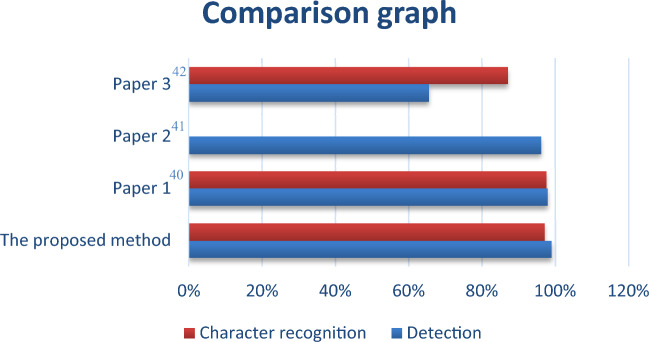
Graphical presentation of the comparison of the proposed method with other techniques.

Figure 13 below is a graphic representation of Table 7.

From the graph above, it’s noticeable that the proposed method outperforms the existing methods for both detection and character recognition.

## Discussion

License plate extraction and recognition is a very promising field of research; many applications tend to implement it for either security or surveillance. However, like any other method, some situations where the proposed technique fell short of expectations were faced, like in the case where images represent low resolution, excessive noise, inadequate lighting, or motion blur. As a result, finding and precisely reading the license plate characters has become difficult. On the other hand, license plates on certain cars might not follow the norms, whether it comes to size, form, or arrangement. These differences may cause the extraction algorithm to become confused, producing unreliable results. Additionally, parts of the license plate may sometimes be obscured by glare from sunshine or reflections from bright surfaces, making it difficult for the recognition algorithm to recognize the characters accurately.

To guarantee reliable performance, a combination of strategies and technologies were employed to address issues such as changing lighting or image quality. To get beyond these challenges, the characters were segmented using the thresholding technique, taking into account changes in brightness in various areas of the image. Additionally, to help differentiate the plate zone from the background by clustering pixels with similar color values, K-means clustering is also used to group pixels within the plate area based on color or intensity similarity. This helps isolate characters from the background. The suggested method's robustness and accuracy were improved by adding morphological processes, especially in situations where the illumination, quality of the image, and character styles varied. Furthermore, touching characters can be separated, or gaps between characters can be closed using operations like opening and closing.

## Conclusion and future work

In this paper, a novel technique for detection and recognition has been proposed, where license plates are used as a use case. The proposed method is a hybridization of deep learning by using the Yolo v8 technique for object detection. For this, a new dataset of 270 images was created, and that was annotated using the CVAT tool. Subsequently, after applying the Yolo v8 method, a bunch of machine learning techniques were used to enhance the extracted license plate. Afterward, the final part of the proposed technique was to identify the country of the car by detecting the character in the license plate that refers to its country. Accordingly, the obtained results were very promising and satisfactory.

Moreover, this technique was tested and verified on different illumination conditions, as well as at different angles at which the image was taken; on the other hand, the suggested method aims to increase accuracy, speed, and adaptability to a variety of real-world scenarios. Therefore, in the upcoming research, more reliable, accurate, and efficient systems will be created with a wide range of applications in security, surveillance, transportation, and other fields. We are planning to develop this technique to be applied to different types of license plates, including plates with different symbols, shapes, or colors. Moreover, future work will address more challenging situations, including images taken from various distances, challenging angles, differing illumination conditions, and occlusion.

### Ethics approval

All authors are contributing and accepting to submit the current work.

### Consent to participate

All authors are contributing and accepting to submit the current work.

### Consent to publish

All authors are accepting to submit and publish the submitted work.

## Data Availability

The data that supports this study is free and the images were downloaded from the following resources that are available in the public domains: [https://www.avito.ma/, https://www.caradisiac.com/, and https://platesmania.com/]. All data are available upon request from the corresponding author.

## Code availability

The code used in this study can be accessed at the following GitHub link: https://github.com/HanaeMoussaoui/License-plate-detection-and-recognition/tree/main.
